# Long-term outcome of 9G MRI-guided vacuum-assisted breast biopsy: results of 293 single-center procedures and underestimation rate of high-risk lesions over 12 years

**DOI:** 10.1007/s11547-024-01808-9

**Published:** 2024-03-21

**Authors:** Giuseppe Rescinito, Nicole Brunetti, Alessandro Garlaschi, Simona Tosto, Licia Gristina, Benedetta Conti, Diletta Pieroni, Massimo Calabrese, Alberto Stefano Tagliafico

**Affiliations:** 1 Department of Radiology, IRCCS - Ospedale Policlinico San Martino, Largo Rosanna Benzi 10, 16132 Genoa, Italy; 2 Department of Experimental Medicine (DIMES), University of Genova, Via L.B. Alberti 2, 16132 Genoa, Italy; 3 Radiology Section, Department of Health Sciences (DISSAL), University of Genova, Via L.B. Alberti 2, 16132 Genoa, Italy

**Keywords:** Biopsy, Breast neoplasms, Image-guided biopsy, Magnetic resonance imaging, Retrospective studies, Vacuum

## Abstract

**Purpose:**

Breast magnetic resonance imaging (MRI) can detect some malignant lesions that are not visible on mammography (MX) or ultrasound (US). If a targeted, second-look fails, MRI-guided breast biopsy is the only available tool to obtain a tissue sample and pathological proof of these “MRI-only lesions”. The aim of this study is to report the performance and underestimation rate of 9G MRI-guided vacuum-assisted breast biopsy (VABB) over 12 years at a single center.

**Material and methods:**

All 9G MRI-VABB procedures performed from January 2010 to December 2021 were retrospectively reviewed. Two MRI scanners (1.5 T and 3 T) were used with the same image resolution and contrast media. All suspicious lesions detected only by breast MRI underwent biopsy. Reference standard was histological diagnosis or at least 1-year negative follow-up. All malignant and atypical lesions underwent surgery, which was used as the reference standard.

**Results:**

A total of 293 biopsies were retrospectively reviewed. Histopathological VABB results revealed 142/293 (48.4%) benign lesions, 77/293 (26.2%) high-risk lesions, and 74/293 (25.2%) malignant lesions. No significant complications were observed. Surgical pathology results allowed for the reclassification of n = 7/48 B3b lesions: n = 4 were ductal carcinoma in situ, while n = 3 presented invasive features at surgical histology (2 IDC; 1 ILC). B3b underestimation occurred overall in 14.6% of B3 cases. Breast follow-up was achieved for all benign VABB results, and only one false-negative case was observed.

**Conclusion:**

Our results confirm that 1.5 T and 3 T MRI-guided VABB is an accurate and safe procedure for histopathologic final diagnosis of MRI-only lesions. Critical issues remain the potential high-risk underestimation rate of B3b VABB results and management of follow-up of benign lesions.

## Introduction

MRI is the best imaging technique to detect breast lesions not identified by mammograms and ultrasound. Breast MRI is highly sensitive but lacks specificity for breast cancer [1–3]. MRI-only lesions are those lesions that are not detected by standard techniques such as mammography (MX), tomosynthesis, and ultrasound (US) and are not even visible by second-look US [3–5]. When a lesion detected only by MRI is considered suspicious, histological verification with MRI-guided VABB is needed and recommended by European guidelines [6]. Several studies demonstrated the feasibility, safety, and accuracy of MRI-guided vacuum-assisted breast biopsies, but cancer detection rates, false negative examinations and underestimation rates have high variability, according to several published data [7–14]. In particular, the underestimation rate of MRI-VABB for high-risk lesions is higher than vacuum-assisted stereotactic and even ultrasound-guided biopsies [15]. MRI-VABB differences across different centers could be partly explained by factors such as lesion location, type of enhancement, post-biopsy management, pre-biopsy planning, choice of the biopsy device, data collection, and even center expertise in general [16]. Regarding VABB needle size, several sizes are provided by different vendors ranging between 8–12 G. At our institution we perform MRI-VABB with a 9 G needle with an average number of 10–12 cores obtained. Therefore, the aim of this study was to report results of 9G percutaneous MRI-VABB for MRI-only detectable breast lesions and to assess the underestimation rate of high-risk lesions (B3) comparing the result to surgical histological examination as a gold standard.

## Materials and methods

The study was approved by the local Ethics Committee. The requirement for patients’ informed consent was waived due to the retrospective nature of the study. All patients who underwent MRI-guided VABB from January 2010 to December 2021 were included.

We consecutively reviewed all VABB procedures performed under MRI guidance at a single center from our institutional database (IRCCS Ospedale Policlinico San Martino, Genova, Italy) between January 2022 and June 2022. We included women with MRI-only visible suspicious lesions that could not be identified by mammography or ultrasound alone, including second-look examination.

Patients were considered for MRI-guided biopsy if they met the following inclusion criteria: MRI lesions categorized as BI-RADS 4–5 with a negative second-look examination and BI-RADS MRI 3 lesions based on patient anxiety or if the patient was scheduled for surgery. We excluded 10 MRI-only lesions because the target had disappeared in the pre-biopsy T1 dynamic 3D T1-weighted gradient-echo sequences.

All MRI VABB procedures were performed by three experienced breast radiologists (each with a minimum of 10 years of breast ultrasound experience, reading at least 4000 mammograms annually, and performing over 400 VABBs per year for the past decade). For benign lesions, up to December 2021, a six-month follow-up MRI and then annual mammography/ultrasound were recommended. All patients with a diagnosis of malignancy underwent surgery within 30 days of biopsy. The standard of reference was established by at least 18-months follow-up for benign biopsy results. For cases with malignant or high-risk results, the standard of reference was determined based on surgical histology results.

### MRI Vacuum-assisted breast biopsy-freehand procedure

All biopsies were performed on a 3 T GE Signa HDx 3.0 T; General Electric Medical Systems, Milwaukee, WI) and 1.5 T (Symphony, Siemens) machine with a dedicated eight-channel open breast coil. Patients were positioned prone on the MRI table using a dedicated breast biopsy coil with a grid compression device to facilitate lesion localization and prevent motion during the biopsy procedure. A marker with vitamin E capsule was taped to a grid cell near the expected lesion site.

### MRI protocol

In this study, MRI protocol included: 2D sagittal T1-weighted sequence for visualization of the markers inside the compression device, a dynamic 3D T1-gradient- echo weighted sequence and image acquisition before and after gadolinium administration (0.15 mmol/kg of gadobenate dimeglumine (GdBOPTA-Multihance; Bracco Imaging, Milan, Italy) and a 20-mL saline infusion and a 3D T1-weighted sequence to check the position of the localizing device. A subtraction of the unenhanced images was then performed. The lesion coordinates were obtained by placing a cursor over the lesion on the axial and sagittal images and calculating its distance from the fiducial marker along the x-, y-, and z-axes. Needle positioning guidance was then manually obtained based on the spatial relationship between the lesion, the vitamin E marker, and grid lines. All procedures were performed via the lateral approach. We used a biopsy kit (Suros ATEC: Hologic, Marlborough, MA, USA), containing a needle guide, coaxial cannula (made of sterile plastic), stylet (made of titanium) and obturator. The VABBs were performed with a 9-gauge vacuum-assisted biopsy probe (Atec Suros Surgical Systems, Indianapolis, IN, USA). Another T1-weighted sequence was repeated to document the location of the obturator. After the stylet was replaced with a sterile plastic MRI-visible obturator, the imaging was used to confirm that the obturator was correctly positioned relative to the lesion. Once this was confirmed, the obturator was removed and replaced with the VAB device, and biopsies were collected. The median number of VABB specimens obtained was 10 (range 6–18). At the end of sampling, an MRI-compatible titanium clip was released at the biopsy site (Atec TriMark, Hologic, Indianapolis, IN, USA). Prior to clip positioning, a sequence was performed to visualize the biopsy site and detect any potential complications, such as hematoma.

**Fig. 1:**
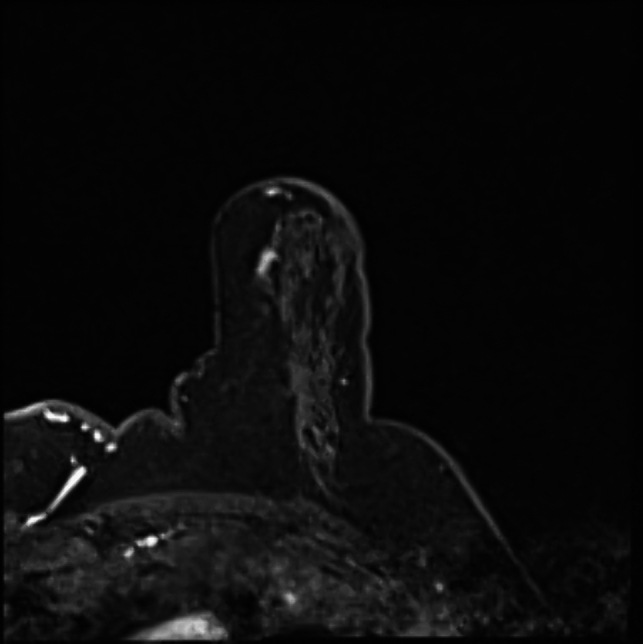
Example of an MRI VABB on 1.5 T scanner that shows a small non-mass-like enhancement in the left breast. Upon histological examination, invasive lobular carcinoma was diagnosed

**Fig. 2:**
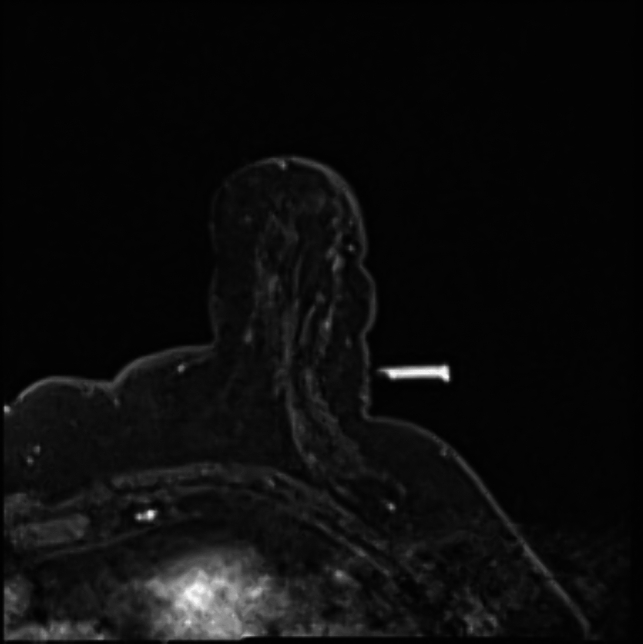
Vitamin E capsule as fiducial marker localized in a T1 axial sequence

**Fig. 3:**
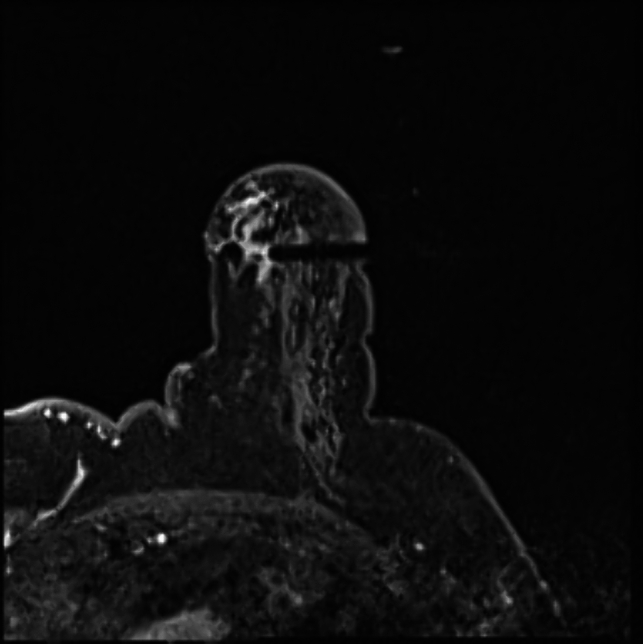
The target introducer and the lesion are lined up

**Fig. 4:**
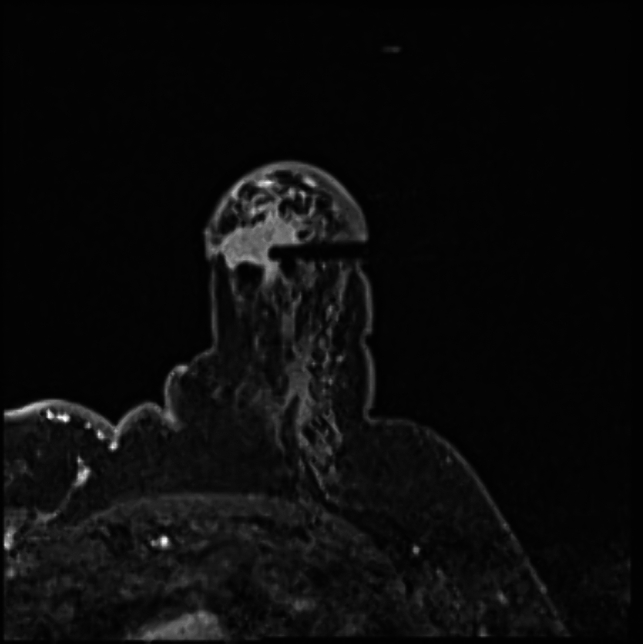
The T1-weighted sequence performed at the end of the sampling procedure reveals the presence of a hematoma at the biopsy site

Figures 1, 2, 3 and 4 illustrates the complete procedure.

### Pathological and radiological correlation

Two pathologists with more than 15 years of experience in breast pathology were involved in assessing of the VABB’s specimens. The NHS Breast Cancer Screening program classifies breast lesions into the following categories: B1 normal tissue or non‑diagnostic, B2 benign, B3 lesion with uncertain malignant potential, B4 suspicious for malignancy or B5 malignant tissue [17].

B3 lesions were further classified into the following categories according to the World Health Organization classification: atypical ductal hyperplasia (ADH); flat epithelial atypia (FEA); lobular neoplasia, including atypical lobular hyperplasia (LIN1) and classical lobular carcinoma in situ (LIN2); radial scar or complex sclerosing lesion (RS-CSL); papillary lesions (PL) and phyllodes tumor (PT) [18]. Additionally, all B3 lesions were subclassified into B3a or B3b lesions based to cytologic-histologic atypia [19]. A radiologist with more than 25-years of experience in breast imaging assessed the concordance between the pathology results and radiological findings. Every case underwent multidisciplinary discussion, and every B3b lesion underwent surgery. In the case of B3a lesions, patients undergo surgery based on either the recommendation of the surgeon or patient preferences. Before surgery, stereotactic or ultrasound localization was performed on the post-VABB hematoma in cases where the target lesion was completely removed or located near the non-migrated clip. B3 lesions were considered “upgraded” if malignancy, such as invasive carcinoma or ductal carcinoma in situ, was reported in the final pathology result.

### Statistical analysis

Descriptive statistics was used. Upgrade rates were calculated using surgery as reference standard. Direct and stratified comparisons of upgrade rates among subgroups were conducted with the Fisher’s exact test when appropriate. Analyses were performed using STATA 15 MP (StataCorp LLC 4905 Lakeway Drive College Station, Texas 77,845–4512, USA). *P* value < 0.05 was considered statistically significant.

## Results

A total of 293 MRI-VABB procedures were performed at our institution between January 2010 and December 2021. MRI-guided VABB was not performed in 10/303 patients (3.3%), due to the overly posterior locations of the lesions.

VABBs were successfully conducted for 293 MRI-only visible suspicious lesions that could not be identified by MX and US.

The mean age of the patients was 56.5 years, ranging from 21 to 87 years.

The average size of the analysed lesions using VABB was 14.5 mm, ranging from 4 to 70 mm. No clinically significant bleeding or major patient discomfort was reported during the procedures.

Table 1 provides the characteristics of the 293 analyzed lesions.

**Table 1:** Imaging and histological characteristics of diagnosed lesions at our center

Characteristic	Number of patients
*Histological subtype*	
B2	142 (48, 4%)
B3a	29 (9, 9%)
B3b	48 (16, 3%)
B4	1 (0, 3%)
B5	
Ductal carcinoma in situ (DCIS)	20 (6, 8%)
Invasive ductal carcinoma (IDC)	36 (12, 3%)
Invasive lobular carcinoma (ILC)	16 (5, 5%)
Other	2 (0, 6%)
*Lesions size*	
≤ 10 mm	147 (50, 2%)
11–19 mm	83 (28, 3%)
≥ 20 mm	63 (21, 5%)
*Enhancement pattern*	
Mass-like	173 (59%)
Non-mass like	120 (41%)
*MRI-BIRADS*	
BIRADS 3	16 (5, 5%)
BIRADS 4	256 (87, 3%)
BIRADS 5	21 (7, 2%)
*MRI-VABB indications*	
Screening of high risk patients	41 (14%)
Preoperative staging	133 (45, 4%)
Screening of intermediate-risk women	42 (14, 3%)
Problem solving	44 (15%)
Neoadjuvant chemotherapy	7 (2, 4%)
Nipple discharge	4 (1, 4%)
Carcinoma of unknown primary origin	6 (2%)
Suspected recurrence on the scar	16 (5, 5%)

During the pathology examination, only one B1 result was encountered, which required a single repeat procedure. On final histological examination, one B4 lesion was reclassified as a B3b lesion (ADH).

No statistically significant differences were observed in the malignancy rate between mass-like and non-mass-like lesions (*p*-value = 0.14).

Long-term follow-up data was available for 273/293 patients (93, 1%).

At our institution, until January 2021, all patients after VABB underwent MRI follow-up 6 months after the biopsy.

Subsequently, high-risk patients (BRCA +) were further monitored with MRI, while the remaining patients were monitored using conventional methods such as MX and US. We lost n = 20 patients (6.8%) after the initial MRI follow-up at 6 months post-biopsy. The average follow-up period for our cases was 81 months, ranging from 18 months to 12 years. Given these limitations, our study reported only 1 case (0.4%) of false-negative results. This case was identified through the first 6-month MRI follow-up, as the target lesion showed growth in size. Subsequently, a second MRI VABB was performed, revealing a small invasive ductal carcinoma (IDC).

All patients with high-risk (B3b), suspicious (B4) and malignant lesions (B5) underwent surgery. Surgical excisions were performed using mammographically guided needle localization of the metallic clip deployed during the final step of the MRI-guided biopsy. No cases of clip migrations were observed.

All these results were confirmed by final histological examinations underlining the reliability of MRI VABB procedure.

The VABB examination underestimated the B3b final histological results in 7 cases, resulting in a B3b underestimation rate of 14.6%. Among these 7 B3 lesions, 6/7 (85, 7%) B3b lesions were ADH, while 1/7 was a LIN 1–2, as indicated in Table 2.

**Table 2:** Upgrade rate of B3 lesions in our experience

Lesions	Subtypes	Number	Surgery	Upgrade	Rate (%)
B3a	PL	4	1	0	0
	CSL	7	2	0	0
	UDH	17	0	0	0
	RS	1	1	0	0
B3b	FEA	10	9	0	0
	ADH	26	26	6	23, 1
	LIN(1–2)	11	8	1	9, 1
	PA	1	1	0	0

PL papillary lesions, CSL complex sclerosing lesion, UDH usual ductal hyperplasia, RS radial scar, FEA flat epithelial atypia, ADH Atypical ductal hyperplasia, LIN 1 Atypical lobular hyperplasia, LIN 2 lobular carcinoma in situ, PA papilloma with atypia

The high-risk lesions that were upgraded had an average size of 12.6 mm (range: 5–20 mm). None of the cases initially classified as FEA were upgraded to malignant lesions. Similarly, there were no instances where ductal carcinoma in situ (DCIS) was upgraded to invasive cancer. Furthermore, no upgrades to carcinoma were observed in the MRI-guided biopsies for B3 lesions without atypia (B3a). Complete data are presented in – 2.

### Literature review

In January 2023, a literature review was performed on MEDLINE (PubMed, https://www.ncbi.nlm.nih.gov/pubmed/).

The search string used were “MRI-guided breast biopsy” AND “high-risk lesions”.

The search was limited to original articles published in English within the past twelve years. Two independent readers (N.B. and B.C. with 5- and 2-year experience in breast imaging respectively) performed the article research. Any disagreements were resolved through consensus. We identified a total of 49 articles published in the last twelve years during our literature review. However, we only included articles that reported the frequency of B3 lesion upgrades. After excluding 43 articles due to incomplete data, our review was narrowed down to six articles. A flowchart illustrating the study selection process can be found in Fig. 4.

The upgrade rate of B3b lesions, as reported in the articles included in our literature review, is presented in Table 4.

## Discussion

The first-line approach for incidentally detected MRI lesions is a targeted second-look examination using MX and US. In most cases, abnormal findings on breast MRI, particularly those with a high risk of malignancy and mass-like lesions, can be identified through experienced physicians conducting second-look examinations using US and MX [4]. However, in cases where suspicious lesions lack corresponding findings on US/MX, there is still a low but significant probability of malignancy. Therefore, MRI-guided biopsy is necessary to investigate such lesions [11, 12].

In our study, our primary objective was to calculate the diagnostic accuracy of 9G percutaneous MRI-VABB specifically for detectable breast lesions. Additionally, we aimed to determine the rates of upgrading to malignancy for MRI-VABB-detected breast lesions and assess the corresponding underestimation rates of high-risk lesions. To establish a reliable gold standard, we used the results of the final histological examination. Obtaining data on the risk of malignancy for upgraded B3 lesions is essential for making appropriate management recommendations when high-risk lesions are diagnosed through MRI-VABB.

To the best of our knowledge, a freehand VABB technique has been described in only a few other studies in literature [13, 14]. Our results, especially the rates of benign (48.4%), high-risk (26.2%), and malignant lesions (25.2%), are in line with recent literature [8, 9]. In our report, MRI-guided VABB was not performed in 3.3% of cases, which aligns with the range reported in the literature (3–13%) [15]. This was primarily due to the non-visualization of the target at the grid position, which could be attributed to hormonal changes or indicating a benign lesion. For these patients, management was carried out through MRI follow-up.

A VABB diagnosis of a benign lesion is considered reliable when there is agreement between radiological suspicion and histological results [20, 21]. In the literature, it is recommended to conduct an MRI examination at 6 months after a benign concordant VABB [22, 23]. In contrast, based on our experience, we consider the biopsy result to be adequate without the need for a subsequent MRI 6-month follow-up, depending on factors such as the initial radiological suspicion, imaging findings before and after the biopsy, and concordant histological results. Due to the low rate of false negative results, we recommend a standard annual MX/US follow-up after a benign-concordant MRI VABB. However, it is important to note that, unlike stereotactic and US-guided VABB, real-time confirmation of the removed target lesion is unavailable in MRI-biopsy. Additionally, the presence of a residual lesion cannot be completely ruled out in the biopsy cavity after VABB, as gadolinium is not present and post-procedural bleeding can obscure visibility. A close MRI follow-up is advised in the case of a discordant result from the MRI VABB, as it enables the detection of enhanced residual lesions that may require a second biopsy or further follow-up.

Therefore, based on our current experience in 2023, a 6-month MRI follow-up after a negative VABB is not recommended. We acknowledge that this approach might not be applicable on a broader scale due to the extremely low number of false negative cases. This data could be extended to other hospital settings characterized by a significant number and extensive expertise of MRI-guided VABB biopsies.

The management of B3 lesions remains controversial.

According to the UK guidelines [24], vacuum-assisted excision (VAE) is recommended as the gold standard for managing all B3 lesions without atypia (B3a). Surgical management, on the other hand, is recommended for lesions with atypia (B3b) due to a significantly higher risk of underestimation. In the Third International Consensus Conference on lesions of uncertain malignant potential in the breast (B3 lesions) [25], the majority of panelists suggest open excision for ADH and PT, while for other B3 lesions (RS, FEA, PL, LIN) VAE is considered an alternative to open excision. High-risk lesions that were diagnosed by MRI VABB, and in which a subsequent diagnosis of invasive cancer or DCIS lesion was made at surgical excision were considered underestimates [23]. In our study, no upgrades to carcinoma were observed in the MRI-guided biopsies for B3 lesions without atypia (B3a). Based on these findings, in our institution, we recommend surgical excision for B3a lesions only if it is recommended by the surgeon, based on patient preferences, or if these lesions have a size larger than 15 mm. No cases of FEA and PA have been upgraded to malignant lesions. However, according to Lourenco et al. [26] there is a significant risk of underestimation for RS (23.1%) and PA (5.9%) identified during MRI-guided breast biopsy. Based on our analysis, we recommend surgical intervention for PA lesions as well. In fact, it’s worth noting that we only analyzed one PA lesion in our study. On the other hand, we examined 10 cases of FEA, and all FEA lesions had dimensions less than 10 mm. Based on this observation, it can be concluded that surgery could be avoided for FEA lesions with dimensions below this threshold especially if the MRI VABB procedure was considered adequate.

Our study found an ADH upgrade rate of 23.1%, which falls within the lower range of upgrades reported in the literature (Table 3—16.7–100%) [30–32]. Consistent with our findings, other studies have also demonstrated that ADH is the most commonly identified B3 lesion on MRI and has the highest upgrade rate [33, 34]. Based on these results, we recommend excision of ADH lesions detected on MRI-guided core biopsy. This recommendation is supported by the findings of Michaels et al. [29], who confirmed that ADH lesions are more likely to upgrade compared to other B3 lesions (Table 4).

**Table 3:**
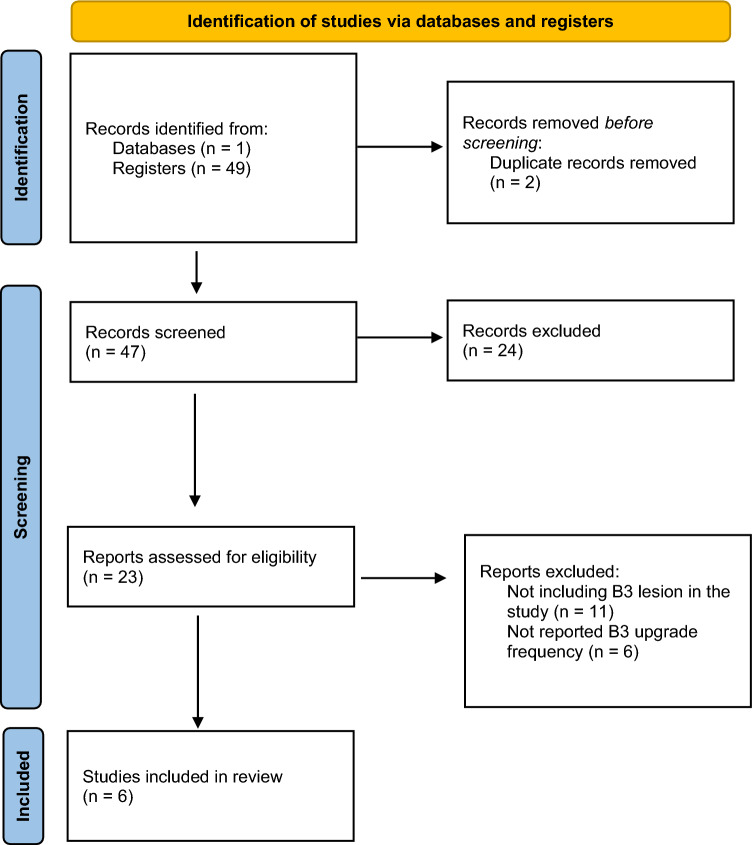
Study selection flow-chart according to the preferred reporting system for systematic reviews

**Table 4:** The upgrade rate of B3b lesions reported in literature

First author	ADH upgrade (%)	LIN upgrade (%)	FEA upgrade (%)	PA upgrade (%)
Laurenco [25]	31.6	28,6	NF	5.9
Okamoto [26]	38.9	29	NF	8
Speer [27]	19	6	NF	NF
Michaels [28]	22.5	6.3	33.3	0
Crystal [29]	100	50	50	NF
Cha [30]	16.7	40	NF	NF
Rescinito	23, 1	9, 1	0	0

*NF* not found

In our experience, we found that LIN1-2 lesions have an upgrade risk of 9.1%, which falls within the lower range reported in other studies in the literature (16%) [29].

Although the upgrade rate of LIN lesions to malignancy is lower compared to ADH, it is still considered high. These results, in line with the existing literature, indicate that B3b lesions detected on MRI-VABB should be considered for surgical excision, particularly in cases of ADH or LIN diagnosis.

In conclusion, our study reveals a relatively high rate of upgrade to malignancy for high-risk lesions, specifically ADH or LIN, detected through MRI-VABB. This finding suggests that surgical excision is warranted for these lesions. It is worth noting that there were no specific MRI imaging characteristics that could reliably predict the upgrade to malignancy, emphasizing the importance of pathology in guiding the decision for surgical excision. Furthermore, our malignancy rate aligns with those reported in the literature, indicating consistency with previous studies (25.2% vs. 21% in other studies) [33].

MRI-guided VABB procedures have proven to be effective in characterizing suspicious non-palpable breast lesions detected exclusively through MRI. Our study demonstrates that these procedures are well-tolerated, simple to perform, and reliable in their results. This consistency in performance allows for the identification of new disease, enabling healthcare professionals to make crucial decisions for accurate surgical planning.

This study has main limitations. The first limitation is its retrospective design.

Additionally, the study was conducted at a single center, which may limit the generalizability of the findings to other settings. Furthermore, we were unable to evaluate the negative predictive value of MRI-guided biopsy due to the loss of some patients during the follow-up period. Another limitation is the small sample size, particularly for each subtype of B3b lesions, which may affect the statistical power and precision of the results. However, by comparing our findings to those of similar studies, we have contributed additional data and information that can help guide the management of B3 lesions detected through MRI.

## Conclusion

Our results confirm that both 1.5 T and 3 T MRI-guided VABB procedures are accurate, cost-effective, and safe methods for obtaining histopathologic diagnosis of MRI-only lesions. However, it is important to note the significant risk of underestimation observed in B3b VABB results, which highlights the necessity for these lesions to undergo surgical intervention for definitive histological diagnosis.

It is crucial to set up a proper follow-up for benign lesion, based on our current experience, a 6-month MRI follow-up after a concordant MRI VABB is not recommended.

In conclusion, our results demonstrate that MRI-guided VABB is a highly reliable technique with a very low long-term false-negative ratio.

## Abbreviations

ADH: Atypical ductal hyperplasia
BI-RADS: Breast imaging reporting and data system
DCIS: Ductal carcinoma in situ
FEA: Flat epithelial atypia
IDC: Invasive ductal carcinoma
ILC: Invasive lobular carcinoma
LIN: Lobular neoplasia
MRI: Magnetic resonance
MX: Mammography
PL: Papillary lesions
PT: Phyllodes tumor
RS-CSL: Radial scar or complex sclerosing lesion
US: Ultrasound
VABB: Vacuum-assisted breast biopsy
VAE: Vacuum-assisted excision
